# Incident HIV infection has fallen rapidly in men who have sex with men in Melbourne, Australia (2013–2017) but not in the newly-arrived Asian-born

**DOI:** 10.1186/s12879-018-3325-0

**Published:** 2018-08-20

**Authors:** Nicholas A. Medland, Eric P. F. Chow, Timothy H. R. Read, Jason J. Ong, Marcus Chen, Ian Denham, Praveena Gunaratnum, Christopher K. Fairley

**Affiliations:** 1 Melbourne Sexual Health Centre, Alfred Health, Melbourne, Australia; 2 Central Clinical School, Monash University, Melbourne, Australia; 3 The Kirby Institute for Infection and Immunity, University of New South Wales, Sydney, Australia; 4 Faculty of Tropical and Infectious Diseases, London School of Hygiene and Tropical Medicine, London, UK

**Keywords:** HIV, Incidence, Treatment as prevention, Pre-exposure prophylaxis, Sexual transmission

## Abstract

**Background:**

We examined differences in incident HIV infection between newly-arrived Asian-born and other men who have sex with men (MSM) after the introduction of universal HIV treatment guidelines in 2015 and pre-exposure prophylaxis in 2016.

**Methods:**

Clinical, demographic, laboratory and behavioural data on MSM presenting for HIV testing at the Melbourne Sexual Health Centre from July 2013 to June 2017 were extracted. We compared the proportion of newly-arrived (four years or less in Australia), Asian-born and other MSM tested each year who were diagnosed with incident HIV infection (negative test within one year or diagnosis with indeterminate or negative Western Blot).

**Results:**

We analysed 35,743 testing episodes in 12,180 MSM, including 2781 testing episodes in 1047 newly-arrived Asian-born MSM. The proportion of other MSM tested each year who were diagnosed with incident HIV infection fell from 0.83% in 2014 to 0.38% in 2017 (*p* = .001), but did not fall in newly-arrived Asian-born MSM (from 1.18% in 2014 to 1.56% in 2017, *p* = .76). In the multivariate logistic regression, in 2016/2017 but not in 2014/2015, being newly-arrived Asian-born was associated with an increased odds of diagnosis of incident HIV infection (aOR 3.29, 95%CI 1.82–5.94, *p* < .001).

**Conclusions:**

The epidemiology of HIV in Melbourne Australia has changed dramatically. While there has been an overall reduction amongst MSM, the incidence of HIV in newly-arrived Asian-born MSM remains high. Failing to address these new inequalities leaves individuals at risk and may offset the population benefit of biomedical HIV prevention.

**Electronic supplementary material:**

The online version of this article (10.1186/s12879-018-3325-0) contains supplementary material, which is available to authorized users.

## Background

The effectiveness of HIV pre-exposure prophylaxis (PrEP) and treatment as prevention (TasP) in interrupting HIV transmission is well established [1, 2]. The scale up of these biomedical prevention modalities is now a central pillar of the HIV response in high income countries [3–5]. HIV incidence may already be declining in urban MSM populations [6]. As with any population intervention, the maximal population benefit will occur with high coverage across the highest risk groups [7]. Incomplete coverage of neglected priority populations may leave individuals at risk and offset the population benefit. In particular, because biomedical HIV prevention is accessed through health care systems, those with less access to health care may benefit less.

In Australia, antiretroviral therapy (ART) is publicly funded and has been available to all patients regardless of CD4 cell count or clinical stage since 2015 [8]. Since 2016, PrEP has been available through large publicly funded projects in major metropolitan centres, including Melbourne [9, 10]. However, eligibility for Medicare, Australia’s national universal health insurance scheme, is required to access antiretroviral therapy nationally and to access PrEP in Melbourne. Melbourne is a so-called fast-track city and reports progress against the 90–90-90 goals of 90%, 94% and 93% of people living with HIV diagnosed, on ART and with virological suppression respectively [11, 12].

The Melbourne Sexual Health Centre (MSHC) in Victoria, Australia, is a large government funded sexual health service which provides free HIV and STI screening to more than 6000 MSM every year and makes more than a quarter of new HIV diagnoses in MSM in the state [13–15]. Unlike other parts of the Australian health care system, sexual health centres are not required to establish patients’ Medicare eligibility and do not charge Medicare ineligible patients for STI testing. However, that funding does not cover the provision of ART or PrEP.

We chose newly-arrived Asian-born MSM as a subpopulation of interest for several reasons. Each year Australia, a country of 24 million people, admits 700,000 temporary migrants (excluding tourists and including approximately 250,000 working holiday makers under 30 years of age) plus more than 700,000 international students [16]. Students from China, India, Nepal, Malaysia and Vietnam make up 53% of the latter group [16]. With some exceptions, temporary migrants and international students are not eligible for Medicare and cannot access publicly funded for ART or PrEP, at least in the state of Victoria [17]. Asian-born MSM may have poorer knowledge of HIV and sexual health or be less successful negotiating risk reduction strategies [18].

This study aims to examine whether there is any difference in the incidence of HIV infection between newly-arrived (i.e. within 4 years) Asian-born and the other MSM and if this has changed since the introduction of of treat-all ART guidelines in 2015 and the widespread availability of PrEP in 2016.

## Methods

We undertook a retrospective observational study by reviewing the records of MSM tested for HIV between July 1st 2013 and June 30th 2017 at MSHC. We included all HIV testing episodes in males of 18 years of age or greater who reported having had sexual contact with other males in the last 12 months. We excluded any testing episode in a patient who had previously received a positive HIV test, including those referred to the centre having recently been tested elsewhere, and also excluded any testing episode in which no date of previous negative HIV test was available, including those presenting for their first test. Patients presenting for care at the MSHC specialist HIV treatment services and patients attending the dedicated clinic for PrEP were not included.

MSHC uses a customised electronic medical record (Clinical Patient Management System, CPMS) which collects demographic, sexual behavioral and clinical information on all patients. All patients complete a self-administered computer-assisted self-interview (CASI) on sexual history such as number and gender of sexual partners, condom use and prior HIV testing. The Melbourne Diagnostic Unit Public Health Laboratory (MDU, University of Melbourne) provides onsite laboratory services for microbiological specimens for *Neisseria gonorrhoeae* (nucleic acid amplification testing (NAAT) and culture) and *Chlamydia trachomatis* (NAAT). The Victorian Infectious Diseases Reference Laboratory (VIDRL) is contracted to perform all off-site laboratory testing including HIV and syphilis serology.

We extracted the following data from the consultation record at which the HIV testing was performed: age, gender, country of birth, year of arrival in Australia, number and gender of sexual partners and condom use in the past 3 months, the date and result of the most recent previous HIV test, if the patient was taking PrEP (recorded from March 2016), symptoms of STI, a new diagnosis of early syphilis. Data provided from MDU included results of testing for gonorrhoea and chlamydia performed at that consultation. Data provided by VIDRL included HIV testing results and prior HIV testing results and syphilis serology. HIV test results were collected from January 1st 2011 so that the inter-test period could be calculated in the vast majority of patients.

Following Australian guidelines, HIV is diagnosed using a two step algorithm: a screening assay which, if reactive, is followed by a confirmatory Western Blot assay [19, 20]. Until April 29th 2014 the screening assay was the Abbott Murex HIV-1 2.0 EIA (3rd generation). After that date the DiaSorin Liaison XL Murex HIV Ab/Ag CLIA (4th generation) assay was used as the screening assay. The MP Diagnostics HIV1/2 Blot 2.0 Western Blot assay was used as the confirmatory test for the entire study period. For this study, the Western Blot was recorded as positive if there were one or more glycoprotein bands and three or more viral protein specific bands. We recorded the result as indeterminate if specific viral bands were present but the above criteria for a positive result were not met. If no viral specific bands were present the assay was recorded as negative.

We defined *incident HIV infection* as confirmed HIV infection with either a recorded previous HIV test within the past 365 days or serological evidence of recent infection (a reactive screening test and a negative or indeterminate Western blot) [21, 22]. To accommodate testing at multiple locations, the date of previous negative HIV test used in the analysis was the most recent of the date recorded in the clinical record on the day of the test and the date of the most recent test performed at MSHC, as has been previously published [22].

Patients were categorized as *Asian-born* if they were born in the following South, East and Southeast Asian countries: Bangladesh, Bhutan, Brunei, Cambodia, China, East Timor, Hong Kong, India, Indonesia, Japan, North Korea, South Korea, Laos, Macau, Malaysia, Maldives, Mongolia, Myanmar, Nepal, Pakistan, Philippines, Singapore, Sri Lanka, Taiwan, Thailand or Vietnam. Patients were categorized as *newly-arrived* if the year of arrival in Australia was 4 years or less before the year of the HIV test. Those that met these criteria on the day of the HIV test were classified as newly-arrived Asian-born MSM.

Study periods were the year from July 1st 2013 until June 30th 2014 (hereafter referred to as 2014) and then the year to June 30th 2015, 2016 and 2017 respectively (hereafter referred to as 2015, 2016, 2017). Each study period was exactly one calendar year.

Our primary outcome measure was the proportion of MSM tested each year (2014–2017) who were diagnosed with *incident HIV infection*. The secondary outcome measure was the proportion of individuals tested each year who were diagnosed HIV positive. For a supplementary analysis to support the choice of study population stratification, we performed analysis of the primary outcome measure in the following population subgroups using the definitions above: newly-arrived Asian-born MSM, newly-arrived not Asian-born MSM and Asian-born not newly-arrived MSM and compared them to MSM not in that subgroup.

Our hypothesis was that a difference between population subgroups would emerge with the introduction of biomedical HIV prevention. Factors that were associated with the odds of diagnosis of incident HIV were examined using logistic regression performed using generalized estimating equation (GEE) models for repeated measures in individuals. The analysis was performed twice covering testing during two time periods: 01 July 2013 to 30 June 2015 and 1 July 2015 to 30 June 2017. We examined the following co-variates in each: age, country of birth (Australia, Organisation for Econonic Development (OECD) member country, Asia as defined above), new arrival in Australia (year of arrival less than 4 years before the year of the test), being newly-arrived Asian-born, time since prior HIV test, reporting consistent condom use (or no anal sexual partners) in the previous 3 months, reporting ten or more sexual partners in the previous 3 months, presentation with symptoms of a sexually transmitted infection on the day of the test, a diagnosis of secondary or recently acquired syphilis, pharyngeal, urethral or anorectal gonorrhea, or urethral or anorectal chlamydia. Covariates associated with the outcome with a *p* value less then.20 were included in the multivariate analysis. Newly-arrived Asian-born status was entered in the multivariate analysis in both models.

All analyses were conducted using the STATA Statistics software package (version 14). This study was approved by the Alfred Hospital Ethics Committee (approval number 335/17). This study is reported in accordance with STROBE and RECORD statements [23, 24].

## Results

We included 2781 HIV tests in 1047 newly-arrived Asian-born men who have sex with men (MSM) and 32,962 HIV tests in 11,133 other MSM performed between 1 July 2013 and 30 June 2017 after excluding first tests in 825 newly-arrived Asian born MSM and 2051 other MSM with no record of prior testing. Compared to other MSM, newly-arrived Asian-born MSM were younger (median 26.4 vs 29.5 years of age, *p* < .0001), had a shorter interval between HIV tests (median 109 vs 133 days, *p* < .0001), were less likely to present with symptoms (16.8% vs 22%, *p* < .001). They were more likely to report consistent condom use and less than ten sexual partners in the previous 3 months. Despite this, these individuals were more than twice as likely to be diagnosed HIV positive (3.53% vs 1.56%, *p* < .001) or with incident HIV infection (2.42% vs 1.13%, *p* < .001). See Table 1.

**Table 1:** Characteristics of men who have sex with men (MSM) undergoing HIV testing at Melbourne Sexual Health Centre (Australia) from July 1st 2013 until June 30th 2017

	Other MSM	Newly arrived Asian born MSM^1^	*P* value
Number of people tested	11,133^2^	1047^4^	
Number of testing episodes^5^	32,962	2781	
Age, years median (IQR)	29.5 (25.0–37.0)	26.4 (23.5–29.8)	<.0001§
Born in Australia, n (%)	6723 (60.4%)	–	–
Born OECD Country^6^	8727 (78.4%)	58 (5.54%)	<.001^¶^
Born S, E or SE Asia^7^	1331 (12.0%)	–	
Newly Arrived^2^	1846 (16.6%)	–	
Days since prior test, median (IQR)	133 (78–273)	109 (63–214)	<.0001§
Symptoms^8^, n (%)	7260 (22.0%)	466 (16.8%)	<.001^¶^
Syphilis^8^, n (%)	515 (1.56%)	44 (1.58%)	.94^¶^
Pharyngeal gonorrhoea^8^, n (%)	468 (1.42%)	23 (0.83%)	.01^¶^
Urethral gonorrhoea ^8^, n (%)	774 (2.35%)	43 (1.55%)	.007^¶^
Urethral chlamydia ^8^, n (%)	897 (2.72%)	62 (2.23%)	.12^¶^
Anal gonorrhoea^8^, n (%)	1508 (4.57%)	130 (4.67%)	.81^¶^
Anal chlamydia^8^, n (%)	1991 (6.04%)	224 (8.05%)	<.001^¶^
Consistent condom use^8^, n (%)	13,975 (46.3%)	1357 (53.9%)	<.001^¶^
10 or more partners in 3 months^8^ n (%)	4501 (13.7%)	233 (8.4%)	<.001^¶^
Positive HIV tests, n	175	36	
% of tests performed^9^	0.53	1.29	<.001^¶^
% of people tested^10^	1.56%	3.53%	<.001^¶^
Incident HIV, n^11^	126	24	
% positive tests^12^	72.0%	66.7%	.52^¶^
% tests performed^9^	0.38	0.87	<.001^¶^
% of people tested^10^	1.13%	2.42%	<.001^¶^

^1.^ Defined as year of arrival in Australia 4 years or less than the year of the HIV test and country of birth one of 26 Eastern, Southern and South-eastern Asian countries

^2.^ Number of individuals who did not meet criteria at any time during the study

^4.^ Number of individuals who met the criteria at any time during the study period

^5.^ Number of tests in people meeting criteria at the time of the test

^6.^ Country of birth given as one of 35 member countries of the Organisation for Economic Development

^7.^ Country of birth given as one of 26 southern, eastern or South-eastern Asian countries

^8.^ Testing episodes

^9.^ Proportion of all included HIV tests performed during the study period

^10.^ Proportion of all included individuals tested during the study period

^11.^ Reported negative HIV test within 1 year or serological evidence of recent infection

^12.^ Proportion of all positives tests which were acute or early

¶ = chi squared

§ Wilcoxon rank-sum (Mann-Whitney) test

In study year 2014 there was no significant difference in consistent condom use reported between the two groups (57.4% in newly-arrived Asian-born MSM vs 53.4% in other MSM in study year 2014 *p* = .12), but it fell in both groups during the study. The fall was greater in other MSM so that by the end of the study period a greater proportion of newly-arrived Asian-born MSM were reporting consistent condom use (51.9% vs 38.2% *p* < .001). Newly-arrived Asian-born MSM were less likely to report 10 partners or more in the previous 3 months in all study periods with no significant change in either group over the study period. In study year 2014, newly-arrived MSM were more likely to be diagnosed with anorectal chlamydia (7.58% vs 4.84%, *p* = .014). However, during the study, anorectal chlamydia increased in other MSM but not in newly-arrived Asian-born MSM, so that by 2017 there was no significant difference between the two groups (7.82% of newly-arrived Asian-born MSM presentations vs 7.07% other MSM, *p* = .35). In 2017, newly-arrived Asian-born MSM were less likely to be taking PrEP (6.8% vs 11.1%, *p* < .001). See Table 2.

**Table 2:** Characteristics of MSM undergoing HIV testing at MSHC in each study year (2014–2017)

	July 2013 to June 2014			July 2014 to June 2015			July 2015 to June 2016			July 2016 to June 2017			P_trend_ 2014–2017	
	Newly-arrived Asian-born MSM^a^	Other MSM	p	Newly arrived Asian born MSM^a^	Other MSM	p	Newly arrived Asian born MSM^a^	Other MSM	p	Newly arrived Asian born MSM^a^	Other MSM	p	Newly arrived Asian born MSM^a^	Other MSM
Individuals tested, n	255^b^	3,988^c^		291^b^	4422^c^		402^b^	5185^c^		581^b^	5608^c^			
HIV tests^d^, n	409	6365		489	7212		745	8923		1138	10,462			
Age in yrs., median (IQR)^e^	26.3 (23.5–30.0)	30.1 (25.3–37.8)	<.0001^m^	26.7 (23.4–30.7)	30.4 (25.6–38.0)	<.0001^m^	26.6 (23.5–29.8)	30.4 (25.7–38.2)	<.0001^m^	26.7 (24.0–30.0)	30.3 (25.6–37.5)	<.0001^m^	.79^n^	.07^n^
Days since previous HIV test, median (IQR)	133 (75–292)	154 (86–316)	.017^m^	116 (72–234)	144 (83–296)	<.0001^m^	106 (57–21)	131 (78–270)	<.0001^m^	114 (73–238)	101 (61–189)	<.0001^m^	<.001^n^	<.001^n^
PrEP use tests n^f,g^ (%)	–	–	–	–	–	–	7 (3.4%)	79 (2.7%)	.55^l^	39 (5.9%)	721 (10.7%)	<.001^l^	.22^n^	<.001^n^
PrEP n persons^f,h^ (%)	–	–	–	–	–	–	7 (3.5%)	72 (2.7%)	.50^l^	33 (6.8%)	542 (11.1%)	<.001^l^	.19^n^	<.001^n^
Tests with:														
Symptoms n^g^ (%)	71 (17.4%)	1549 (24.3%)	.001^l^	81 (16.6%)	1676(23.2%)	.001^l^	119 (16.0%)	1976(22.2%)	<.001^l^	195(17.1%)	2059(19.7%)	.039^l^	.98^n^	<.001^n^
Syphilis^g^ n (%)	3 (0.73%)	78 (1.23%)	.38^l^	6(1.23%)	117 (1.62%)	.50^l^	16 (2.15%)	159 (1.78%)	.47^l^	19 (1.67%)	161 (1.54%)	.73^l^	.19^n^	.16^n^
Pharyngeal gonorrhoea^g^n (%)	6 (1.47%)	106 (1.67%)	.76^l^	7(1.43%)	176 (2.44%)	.16^l^	6 (0.81%)	118 (1.32%)	.23^l^	4 (0.35%)	68 (0.65%)	.23^l^	.012^n^	<.001^n^
Urethral gonorrhoea^g^n (%)	8 (1.96%)	145 (2.28%)	.67^l^	4(0.82%)	192 (2.66%)	.012^l^	19 (2.55%)	198 (2.22%)	.56^l^	12 (1.05%)	239 (2.28%)	.007^l^	.46^n^	.55^n^
Urethral chlamydia^g^n (%)	8 (1.96%)	159 (2.50%)	.49^l^	6 (1.23%)	194 (2.69%)	.049^l^	20 (2.75%)	246 (2.75%)	.91^l^	28 (2.46%)	298 (2.85%)	.45^l^	.27^n^	.19^n^
Anal gonorrhoea^g^ n (%)	11 (2.69%)	180 (2.83%)	.97^l^	17 (3.48%)	283 (3.92%)	.62^l^	41 (5.50%)	510 (5.72%)	.81^l^	61 (5.36%)	535 (5.11%)	.72^l^	.017^n^	<.001^n^
Anal chlamydia^g^ n (%)	31 (7.58%)	308 (4.84%)	.014^l^	37 (7.57%)	315 (4.37%)	.001^l^	67 (8.99%)	628 (7.04%)	.047^l^	89 (7.82%)	740 (7.07%)	.35^l^	.83^n^	<.001^n^
100% condom use^g,i^n (%)	226 (57.4%)	3229 (53.4%)	.12^l^	255 (56.3%)	3405 (50.5%)	.016^l^	354 (53.3%)	3804 (47.0%)	.002^l^	522 (51.9%)	3537 (38.2%)	<.001^l^	.044^n^	<.001^n^
10 or more partners^g,i^n (%)	39 (9.54%)	852 (13.4%)	.026^l^	36 (7.36%)	983 (13.6%)	<.001^l^	48 (6.44%)	1216 (13.6%)	<.001^l^	110 (9.67%)	1450 (13.9%)	<.001^l^	.54^n^	.43^n^
Positive HIV tests^j^, n	4	48		7	48		11	41		14	38			
% tests^g^	0.98%	0.75%	.62^l^	1.43%	0.67%	.052^l^	1.48%	0.46%	<.001^l^	1.23%	0.36%	<.001^l^	0.86^n^	<.001^n^
% people tested^h^	1.57%	1.20%	.61^l^	2.41%	1.09%	.042^l^	2.74%	0.79%	<.001^l^	2.41%	0.68%	<.001^l^	.53^n^	.02^n^
Incident HIV^k^, n	3	33		5	39		7	33		9	21			
% positives^i^	75.0%	68.8%	.80^l^	71.4%	81.3%	.54^l^	63.6%	80.5%	.24^l^	64.3%	55.3%	.56^l^	.62^n^	.22^n^
% tests^f^	0.73%	0.52%	.85^l^	1.02%	0.54%	.11^l^	0.94%	0.37%	<.001^l^	0.79%	0.20%	<.001^l^	.86^n^	<.001^n^
% people tested^g^	1.18%	0.83%	.56^l^	1.73%	0.88%	.15^l^	1.76%	0.64%	.011^l^	1.56%	0.38%	<.001^l^	.76^n^	.001^n^
(95%CI)	(0.38–3.60%)	(0.59–1.17%)		(0.72–4.09%)	(0.65–1.21%)		(0.84–3.65%)	(0.45–0.90%)		(0.81–2.98%)	(0.25–0.58%)			

^a.^ Born in one of 26 South, East and Southeast Asian countries and year of arrival in Australia 4 years or less than the year of the HIV test

^b.^ Number of individuals who met the criteria at any time during that year

^c.^ Number of individuals did not meet the criteria at any time during the study period

^d.^ HIV tests with a known date of previous negative HIV test only were included

^e.^ Age on the day of the first HIV test during the year

^f.^ Patients were ask if they were taking PrEP from March 2016

^g.^ Number and proportion of tests or testing episodes meeting criteria at the time of the test during the study year

^h.^ Number and proportion of individuals tested meeting criteria at any time during the study year

^i.^ Sexual behaviour as reported in the three months prior to testing

^j.^ Confirmed HIV infection in an individual with no prior known positive HIV test

^k.^ Diagnosis with a negative test in the past 365 days or diagnosis with negative or indeterminate Western blot

^l.^ chi squared

^m.^ Wilcoxon rank-sum (Mann-Whitney) test

^n.^ P trend

**Fig. 1:**
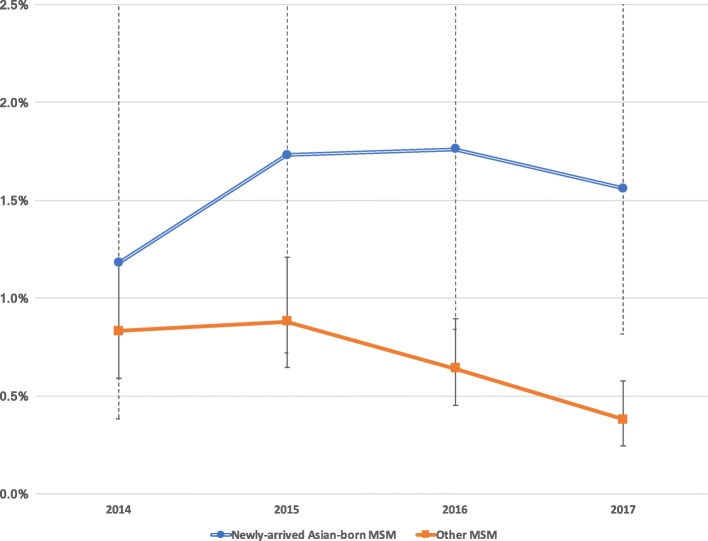
Proportion of individuals tested in each year who are diagnosed with incident HIV infection (2014–2017)

In the first two study years (2014–2015), there was no significant difference between newly-arrived Asian-born and other MSM in the proportion tested each year who were diagnosed with incident HIV infection. However, in the final two study years (2016–2017) a difference emerged and the proportion of newly-arrived Asian-born MSM tested each year who were diagnosed with incident HIV infection was significantly higher than in other MSM. By study year 2017, the proportion of newly-arrived Asian-born MSM diagnosed with incident HIV infection was more than four times other MSM (1.56% vs 0.38% *p* < .001). There was a statistically significant downward trend in other MSM (from 0.83% in 2014 to 0.38% in 2017, p_trend_ = .001). However, the proportion of newly-arrived Asian-born MSM tested each year who were diagnosed with incident HIV infection did not change significantly during the study period (from 1.18% in 2014 to 1.56% in 2017, *p* = .76) See Table 2 and Fig. 1.

In the first two study years (2014–2015), there was no significant difference between newly-arrived Asian-born and other MSM in the proportion of individuals tested each year who were diagnosed HIV positive. However, in the final two study years (2016–2017) a difference emerged and the proportion of newly-arrived Asian-born MSM tested each year who were diagnosed HIV positive and it was significantly higher than in other MSM. By 2017, the proportion of newly-arrived Asian-born MSM tested each year who were diagnosed HIV positive was more than three times other MSM (2.41% vs 0.68%, *p* < .001). There was a statistically significant downward trend in other MSM (from 1.20% in 2014 to 0.68% in 2017, *p* = .02). However, the proportion of newly-arrived Asian-born MSM tested each year who were diagnosed HIV positive did not change significantly during the study period (from 1.57% in 2014 to 2.41% in 2017, *p* = .53) See Table 2.

For the supplementary analysis, in study years 2014 and 2015, we found that there was no statistically significant difference in the proportion tested each year who were diagnosed with incident HIV infection in the following subgroups when compared to the rest of the MSM population excluding that subgroup: newly-arrived Asian-born MSM, Asian-born not newly-arrived MSM, newly-arrived not Asian-born MSM and not newly-arrived not Asian-born MSM. By study year 2017, only the newly-arrived Asian-born subgroup was statistically significantly different from the remaining MSM community. See Additional file 1: Table S1 and Additional file 2: Figure S1.

Being newly-arrived and Asian-born was associated in the multivariate analysis with the odds of being diagnosed with incident HIV infection in the period July 2015–June 2017 (aOR 4.40 (2.38–8.15), *p* < .001)) but not in the period July 2013–June 2015. Consistent condom use and number of sexual partners greater than ten was associated in the earlier time period but not in the later time period. See Table 3.

**Table 3:** Logistic regression analysis, using generalized estimating equations for multiple observations in individuals, on odds of being diagnosed with incident HIV infection in the time period 1 July 2013 to 30 June 2015 and 1 July 2015 to 30 June 2017

	July 2013–June 2015				July 2015–June 2017			
	OR^1^	p	aOR^2^	p	OR^1^	p	aOR^2^	p
Age years^3^	0.77 (0.60–0.98)	.036	0.93 (0.72–1.20)	.56	0.8 (0.68–1.12)	.27		
Not newly-arrived not Asian-born	ref				ref			
Newly-arrived Asian-born	1.79 (0.85–3.79)	.13	1.51 (0.63–3.62)	.36	3.92 (2.14–7.18)	<.001	4.40 (2.38–8.15)	<.001
Asian-born not newly arrived	1.06 (0.53–2.09)	.88			2.43 (1.31–4.51)	.005	2.63 (1.41–4.93)	.002
Newly-arrived not Asian-born	1.44 (0.77–2.71)	.26			1.52 (0.70–3.31)	.29		
Months since prior test	0.97 (0.94–1.00)	.054	0.98 (0.95–1.01)	.12	0.99 (0.96–1.01)	.35		
Symptoms n (%)^7^	1.79 (1.13–2.84)	.013	1.41 (0.84–2.36)	.80	1.37 (0.80–2.34)	.25		
Syphilis n (%)^7^	1.83 (0.45–7.49)	.40			5.72 (2.46–13.3)	<.001	4.01 (1.68–9.55)	.002
Pharyngeal gonorrhoea^7^	1.89 (0.59–6.01)	.28			1.57 (0.22–11.4)	.65		
Urethral gonorrhoea^7^	2.80 (1.13–6.97)	.027	0.86 (0.28–2.63)	.80	1.33 (0.36–5.44)	.69		
Urethral chlamydia^7^	2.05 (0.75–5.63)	.16	1.30 (0.44–3.79)	.63	1.05 (0.26–4.28)	.95		
Anal gonorrhoea^7^	9.41 (5.58–15.9)	<.001	5.97 (3.25–10.97)	<.001	4.09 (2.23–7.49)	<.001	2.97 (1.57–5.63)	.001
Anal chlamydia^7^	4.70 (2.67–8.27)	<.001	2.46 (1.28–4.73)	.007	2.70 (1.45–5.03)	.002	1.69 (0.87–3.27)	.12
100% condom use^8^	0.43 (2.67–8.27)	.001	0.55 (0.33–0.91)	.019	0.57 (0.34–0.95)	.032	0.65 (0.39–1.11)	.12
10 or more partners in 3 months^9^	2.46 (1.50–4.03)	<.001	2.05 (1.21–3.46)	.007	2.09 (1.21–3.62)	.008	1.80 (1.02–3.17)	.04

^1.^ Odds ratio

^2.^ adjusted Odds Ratio

^3.^ Odds ratio per 10 year increment in age

^4.^ Reference group is the rest of the population who is, respectively, not born in Australia, not born in OECD country, not nearly arrived Asian born, not Asian born or not newly arrived

^5.^ Newly-arrived Asian-born was entered in the multivariate analysis instead of separately entering newly-arrived and Asian-born

^7.^ Reference group is the rest of the population/tests who, respectively, did not present with symptoms, were not diagnosed with syphilis, urethral STI or anorectal STI

^8.^ Reports always using condoms in anal sex, or no anal sex, or no sexual partners in past three months. Reference group is those with inconsistent condom use

^9.^ Reference group is those not reporting 10 or more sexual partners in past three months

## Discussion

This study reports the proportion of MSM tested each year who are diagnosed with incident HIV infection at a public sexual health service in Melbourne, Australia from 2013 to 2017 and finds that a difference emerged between newly-arrived Asian-born and other MSM. The proportion tested each year diagnosed with incident HIV infection fell significantly in other MSM but not in newly-arrived Asian-born MSM. Our findings suggest that there was a change in risk factors for incident HIV infection among MSM in Melbourne over the study period. In the beginning of the study period there was no statistically significant difference between the two groups, but by the end of the study period newly-arrived Asian-born MSM were more than four times more likely to be diagnosed with incident HIV infection (1.56% vs 0.38%, *p* < .001). The disappearance of condom use and number of partners in later years as a risk factor for incident HIV suggests that limited access to biomedical interventions in newly-arrived Asian-born MSM is explaining the observed disparity in HIV incidence.

A recently published study of HIV testing in MSM in inner London reports a declining proportion of positive tests from late 2015 to late 2017 which they attribute to early diagnosis, early initiation of treatment and uptake of PrEP, that is biomedical rather than behavioural prevention [6]. Melbourne is a so-called fast-track city and reports progress in excess of 90–90-90 targets [11, 12].

The probability of acquisition of HIV infection is related to the frequency of exposure, the per-exposure probability of transmission and the prevalence of unsuppressed HIV infection in sexual partners. We examined incident HIV infection of less than 1 year duration and therefore assume that the majority of HIV infections included in this study were locally acquired. In this study, newly-arrived Asian-born MSM report less sexual partners and more consistent condom use than other MSM who experienced a falling rate of diagnosis of incident HIV infection despite falling condom use. This suggests a change in vulnerability to HIV infection despite sexual behavior as measured on these indices. Although newly-arrived Asian-born MSM were less likely to report taking PrEP than others, the magnitude of the difference and the overall proportion taking PrEP (6.8% vs 11,1% in 2017 *p* < .001) would not suggest that PrEP use is an explanation for the four-fold difference in incident HIV diagnoses.

Available evidence points toward a substantial decline in the prevalence of unsuppressed HIV infection in Melbourne over the study period. With earlier diagnosis and initiation of antiretroviral therapy, the infectious period between infection and virological suppression has fallen more than five-fold [22]. The magnitude and the timing of these changes could certainly explain the reduction in incident HIV infection in MSM in Melbourne.

Assortative sexual mixing patterns are of increasing interest in HIV transmission research [25]. Our findings could, at least in part, be explained if newly-arrived Asian-born MSM were more likely to have newly-arrived Asian-born MSM as sexual partners and had a higher prevalence of unsuppressed HIV infection. Newly-arrived Asian-born MSM who are overseas students, working holiday makers or temporary migrants will not be eligible for Medicare or publicly funded ART. There is evidence to suggest that individuals born overseas may be less likely to be offered or less likely to initiate early antiretroviral therapy [26, 27]. At our centre, MSM couples have been studied in STI transmission studies [28]. Of MSM couples studied at MSHC, 10 of 42 (24%) newly-arrived Asian born MSM and 30 of 685 other MSM (4.4%) reported being in a relationship with a newly-arrived Asian-born MSM (EPF Chow personal communication: unpublished).

Once diagnosed with HIV infection at Melbourne Sexual Health Centre, patients who are ineligible for Medicare are able to access medical services free of charge including antiretroviral therapy, which is provided through private compassionate access programs supported by the pharmaceutical industry. A recent analysis at this centre showed that country of birth and recent arrival in Australia were not associated with delayed HIV diagnosis or virological suppression after diagnosis [22]. However, this centre is the only publicly funded sexual health centre that is able to offer these services free of charge in a city with a population of approximately five million people and with a large but unknown number of newly-arrived Asian born MSM. Therefore, it is possible that most newly-arrived Asian-born MSM who are infected with HIV are not patients of MSHC. Newly-arrived Asian-born MSM who are not engaged in care at a publicly funded sexual health centre (that provides sexual health and HIV medicine services free of charge) may be more likely to experience a delayed diagnosis or virological suppression after diagnosis.

In fact, delayed HIV diagnosis and virological suppression in newly-arrived Asian-born MSM who are not engaged in care at publicly funded sexual health centres may explain the difference in risk of HIV acquisition observed in this study. The prevalence of undiagnosed and untreated HIV infection (where HIV transmission to others may occur) may be higher in this group than in those with better health care access. Because of sexual mixing patterns, newly-arrived Asian-born MSM may be more likely to have sexual partners who are their peers, that is with peers with are able to transmit HIV infection to others. Sexual mixing patterns and restricted access to HIV testing, HIV treatment and PrEP may explain the large difference in proportion who are diagnosed with incident HIV infection that was observed in this study.

Furthermore, little is known about newly-arrived Asian-born MSM as a population: the size of the population, their sexual behavior, their perception or knowledge of risk of sexually transmitted infections including HIV or how to reduce that risk, how differential health care access might affect outcomes in other areas of health. Rising epidemics of HIV in MSM communities in Asia are increasingly described, including in mainland China [29]. It is not known to what extent these epidemics are connected to HIV transmission amongst newly-arrived Asian-born MSM in Australia.

In this study, we have focused on health care access issues due to their pertinence to biomedical HIV prevention. However, additional research is required to identify and target other vulnerabilities that may place this group at increased risk, which may include language, culture, gay community engagement, sources of HIV information, sexual experience and ability to negotiate successful risk reduction strategies.

Our study is subject to certain limitations. Firstly, the primary outcome measure of our study, the proportion tested each year who are diagnosed with incident HIV infection, is not a direct measure of incidence and may underestimate it. However, the short testing intervals in the study population would suggest that in this population of MSM undergoing frequent retesting that this measure is closely linked to incidence and that there would not be a larger underestimation in other MSM than the newly-arrived Asian born MSM. The large difference in the outcome measure and the small difference in testing frequency between the groups means that a difference in HIV incidence is most probable reason for the observed difference. Secondly, MSM attending MSHC may not be representative of MSM in Victoria. Because patients who are ineligible for Medicare are able to access care free of charge, we would expect newly-arrived Asian-born MSM to be over-represented amongst MSHC patients, making it a useful site for study of this group. Also, because of ease of access at this site, it may be MSM attending MSHC have more frequent HIV testing.

In the era of biomedical HIV prevention, to maximize the success of these costly interventions at the population level, obstacles and barriers to access must be minimized. In Australia, with a highly accessibly universal health care system we have observed that while incident HIV is declining in most MSM, a subpopulation with less access to health care is still experiencing high incidence. Based on this, it is recommended that provision of sexual health and HIV medicine services free of charge, including HIV testing, antiretroviral therapy and preexposure prophylaxis is expanded to cover patients who are not eligible for Medicare. Preventable HIV infections are serious in any subpopulation, but many newly arrived Asian-born MSM will eventually return to countries with poorer protection and services for people living with HIV and may experience stigma, discrimination and poorer health outcomes.

Such intervention would come with considerable costs given that these services are not subsidised by Medicare. However, having a certain subpopulation with higher rates of HIV transmission and lower rates of testing and treatment will undermine the population effect of biomedical HIV prevention and reduce the return-on-investment for treatment as prevention and pre-exposure prophylaxis. This core group of undiagnosed infections will allow HIV to be sustained in the population as they are responsible for a disproportionate amount of transmissions. Hence the benefit of providing services to this group may outweigh the costs.

International students bring more than $20 billion into Australia each year and represent a larger source of foreign income than mining exports [30]. Temporary migrants and international students make up a significant proportion of the population of young sexually active adults and withholding the benefits for accessing PrEP and TasP from them may be an Achilles’ heel of the biomedical prevention approach.

## Conclusion

The epidemiology of HIV in MSM has changed since the introduction of universal HIV treatment recommendations and PrEP and incident HIV infection has fallen dramatically. This fall has created new inequalities which need to be addressed to protect vulnerable individuals from infection and to maximise the overall population benefit. Failing to address inequalities in access to care for newly-arrived Asian-born MSM and other individuals who are not eligible for Australia’s universal health care insurance scheme Medicare may lead to ongoing high rates of HIV transmission in this subpopulation and the wider community. This may threaten Australia’s international reputation as a safe country to visit, work and study.

## Additional files

Additional file 1: **Table S1.** Proportion of individuals tested in each year and diagnosed with incident HIV infection: newly-arrived Asian-born MSM, Asian-born not newly-arrived MSM, newly-arrived not Asian-born MSM and not newly-arrived not Asian-born MSM, compared to the rest of the MSM population not including that subpopulation (DOCX 14 kb)

Additional file 2: **Figure S1.** Proportion of individuals tested in each year diagnosed with acute or early HIV infection: newly-arrived Asian-born MSM, Asian-born not newly-arrived MSM, newly-arrived not Asian-born MSM and not newly-arrived not Asian-born MSM. (PDF 13 kb)
