# Supplementary material for: Incident HIV infection has fallen rapidly in men who have sex with men in Melbourne, Australia (2013–2017) but not in the newly-arrived Asian-born
Source: BMC Infect Dis. 2018 Aug 20;18:410. doi: 10.1186/s12879-018-3325-0 (PMC6102820; doi:10.1186/s12879-018-3325-0)
Supplement: Supplementary file 1 — Table S1. Proportion of individuals tested in each year and diagnosed with incident HIV infection: newly-arrived Asian-born MSM, Asian-born not newly-arrived MSM, newly-arrived not Asian-born MSM and not newly-arrived not Asian-born MSM, compared to the rest of the MSM population not including that subpopulation (DOCX 14 kb) [file 12879_2018_3325_MOESM1_ESM.docx]

**Additional file 1: Table S1 Proportion of individuals tested in each year and diagnosed with incident HIV infection: newly-arrived Asian-born MSM, Asian-born not newly-arrived MSM, newly-arrived not Asian-born MSM and not newly-arrived not Asian-born MSM, compared to the rest of the MSM population not including that subpopulation.**

|  | 2014 | 2015 | 2016 | 2017 | p trend |
| --- | --- | --- | --- | --- | --- |
| Newly-arrived Asian-born MSM | 1.25% | 1.84% | 1.83% | 1.65% | .77^*^ |
| P value | .42^¶^ | .081^¶^ | .004^¶^ | <.001^¶^ |  |
| Asian-born not newly-arrived MSM | 0.98% | 0.82% | 1.48% | 0.48% | .49^*^ |
| P value | .62^¶^ | .87^¶^ | .004^¶^ | .94^¶^ |  |
| Newly-arrived not Asian-born MSM | 1.41% | 0.92% | 0.63% | 0.52% | .06^*^ |
| P value | .11^¶^ | .91^¶^ | .89^¶^ | .80^¶^ |  |
| Not newly-arrived not Asian-born MSM | 0.70% | 0.89% | 0.47% | 0.46% | .005^*^ |

^¶^Chi squared: comparison on that subgroup with the entire population excluding that subgroup
