# Supplementary figures and images for: Incident HIV infection has fallen rapidly in men who have sex with men in Melbourne, Australia (2013–2017) but not in the newly-arrived Asian-born
Source: BMC Infect Dis. 2018 Aug 20;18:410. doi: 10.1186/s12879-018-3325-0 (PMC6102820; doi:10.1186/s12879-018-3325-0)

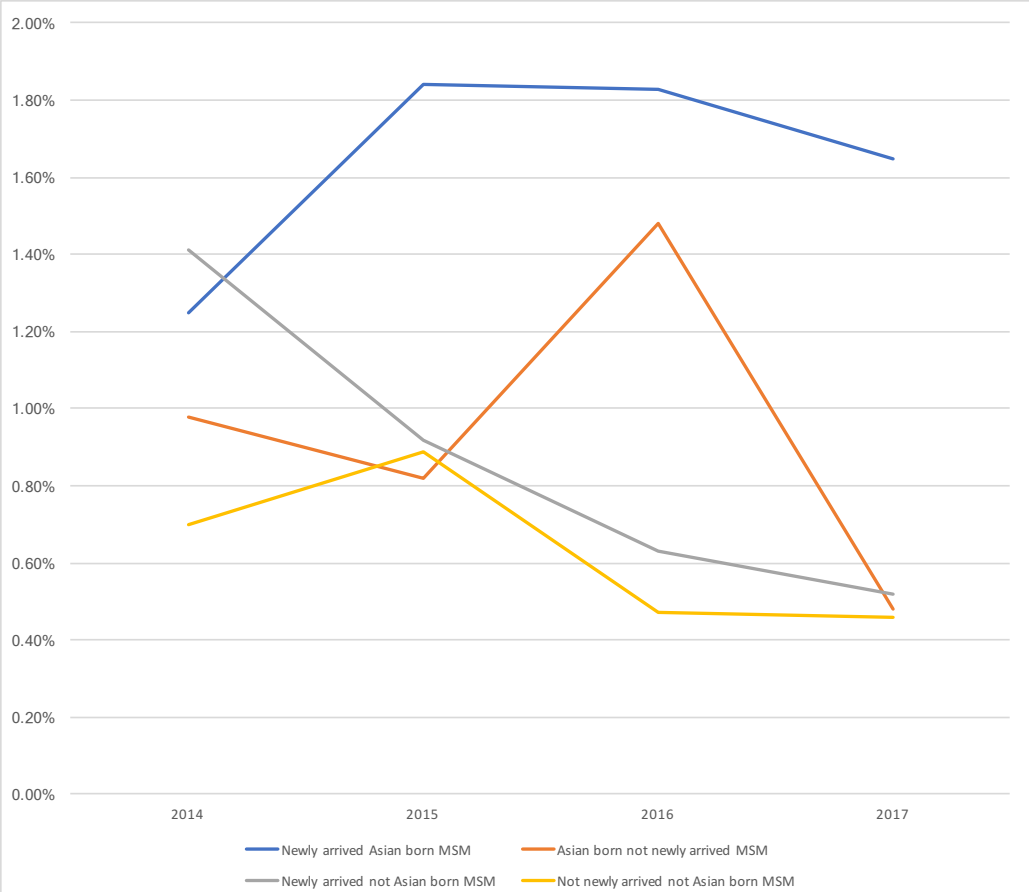

Supplement: Supplementary file 2 — Figure S1. Proportion of individuals tested in each year diagnosed with acute or early HIV infection: newly-arrived Asian-born MSM, Asian-born not newly-arrived MSM, newly-arrived not Asian-born MSM and not newly-arrived not Asian-born MSM. (PDF 13 kb) [file 12879_2018_3325_MOESM2_ESM.pdf]
